# Targeted inhibition of colorectal cancer proliferation: The dual‐modulatory role of 2,4‐DTBP on anti‐apoptotic Bcl‐2 and Survivin proteins

**DOI:** 10.1111/jcmm.18150

**Published:** 2024-03-17

**Authors:** Partha Saha, Mangala Hegde, Kanak Chakraborty, Achinta Singha, Nobendu Mukerjee, Deepshikha Ghosh, Ajaikumar B. Kunnumakkara, Mohd Shahnawaz Khan, Md Irshad Ahmad, Arabinda Ghosh, Ajoy Kumer, Samir Kumar Sil

**Affiliations:** ^1^ Molecular Genetics and Cell Physiology Laboratory, Department of Human Physiology Tripura University Suryamaninagar Tripura India; ^2^ Cancer Biology Laboratory and DBT‐AIST International Center for Translational and Environmental Research (DAICENTER), Department of Biosciences and Bioengineering Indian Institute of Technology (IIT) Guwahati Guwahati Assam India; ^3^ Center for Global Health Research Saveetha Medical College and Hospital, Saveetha Institute of Medical and Technical Sciences Chennai Tamil Nadu India; ^4^ Department of Health Sciences Novel Global Community Educational Foundation Hebersham New South Wales Australia; ^5^ Cell Biology and Physiology Division CSIR‐Indian Institute of Chemical Biology Kolkata West Bengal India; ^6^ Department of Biochemistry, College of Science King Saud University Riyadh Saudi Arabia; ^7^ Department of Structural Biology, School of Medicine UTHEALTH Science Center San Antonio Texas USA; ^8^ Department of Computational Biology and Biotechnology Mahapurusha Srimanta Sankaradeva Viswavidalaya Guwahati Assam India; ^9^ Department of Chemistry, College of Arts and Sciences IUBAT‐International University of Business Agriculture and Technology Dhaka Bangladesh

**Keywords:** 2,4‐Di‐tert‐butylphenol (PubChem CID: 71315956), apoptosis, Bcl‐2, colorectal cancer, mitochondrial stress, Survivin

## Abstract

The anti‐apoptotic proteins, Bcl‐2 and Survivin, are consistently overexpressed in numerous human malignancies, notably in colorectal cancer. 2,4‐Di‐tert‐butylphenol (2,4‐DTBP) is a naturally occurring phenolic compound known for its diverse biological activities, including anti‐cancer properties. The mechanism behind 2,4‐DTBP‐induced inhibition of cell proliferation and apoptosis in human colorectal cancer cells, specifically regarding Bcl‐2 and Survivin, remains to be elucidated. In this study, we employed both in silico and in vitro methodologies to underpin this interaction at the molecular level. Molecular docking demonstrated a substantial binding affinity of 2,4‐DTBP towards Bcl‐2 (Δ*G* = −9.8 kcal/mol) and Survivin (Δ*G* = −5.6 kcal/mol), suggesting a potential inhibitory effect. Further, molecular dynamic simulations complemented by MM‐GBSA calculations confirmed the significant binding of 2,4‐DTBP with Bcl‐2 (dGbind = −54.85 ± 6.79 kcal/mol) and Survivin (dGbind = −32.36 ± 1.29 kcal/mol). In vitro assays using HCT116 colorectal cancer cells revealed that 2,4‐DTBP inhibited proliferation and promoted apoptosis in both a dose‐ and time‐dependent manner. Fluorescence imaging and scanning electron microscopy illustrated the classical features associated with apoptosis upon 2,4‐DTBP exposure. Cell cycle analysis through flow cytometry highlighted a G1 phase arrest and apoptosis assay demonstrated increased apoptotic cell population. Notably, western blotting results indicated a decreased expression of Bcl‐2 and Survivin post‐treatment. Considering the cytoprotective roles of Bcl‐2 and Survivin through the inhibition of mitochondrial dysfunction, our findings of disrupted mitochondrial bioenergetics, characterized by reduced ATP production and oxygen consumption, further accentuate the functional impairment of these proteins. Overall, the integration of in silico and in vitro data suggests that 2,4‐DTBP holds promise as a therapeutic agent targeting Bcl‐2 and Survivin in colorectal cancer.

## 1 INTRODUCTION

Colorectal cancer (CRC) has emerged as a leading oncological challenge, marking its presence as one of the most prevalent gastrointestinal cancers that significantly contributes to global morbidity and mortality.^1^, ^2^ Recent data from GLOBOCAN 2020 elucidate a worrying trend, placing CRC at the third spot in terms of cancer incidence, encompassing 10% of all new cancer diagnoses, while it stands second in causing 9.4% of global cancer‐related fatalities, irrespective of demographic divisions such as age and gender.^3^ The disease exhibits significant variability, influenced by both genetic and environmental factors.^4^ Numerous studies have demonstrated that dietary patterns and inflammation induced by various factors can contribute to the development of CRC.^5^, ^6^ Despite the leaps in oncological diagnostic technologies, the late‐stage diagnosis of CRC remains a persistent issue, leading to complex clinical manifestations.^7^

With an increasing understanding of the cellular and molecular mechanisms underpinning CRC, apoptotic pathways, specifically the targeting of antiapoptotic proteins such as Bcl‐2 and Survivin, have garnered immense attention in therapeutic research.^8^, ^9^ These proteins often showcase elevated expression levels in chemo‐resistant cancer cell subtypes, creating a formidable barrier against the apoptotic potential of many chemotherapeutics.^10^, ^11^ Delving into the specificity of their expression, Bcl‐2 is found to be predominantly expressed in intestinal stem cells (ISCs) across both human and rodent models.^12^ Prior studies highlight the pivotal role of Bcl‐2 in safeguarding ISCs from radiation‐mediated injuries; intriguingly, the pharmacological inhibition of Bcl‐2 manifests retardation in the proliferation of malignant cells, accentuating its potential as a chemopreventive target.^12^ Parallelly, Survivin, identified as the most compact member of the inhibitor of apoptosis protein (IAP) family, presents an overexpression pattern across an array of cancers, including CRC, with its presence being minimal in mature, differentiated tissues.^13^ Given these unique expression profiles, both Bcl‐2 and Survivin have emerged as compelling targets for the development of innovative CRC therapeutics.

Nevertheless, the clinical management of CRC is riddled with challenges, most prominently due to inherent drug resistance mechanisms and a myriad of other molecular complexities.^14^ In this therapeutic quest, nature's vast arsenal of molecules has often been the starting point for the identification of efficacious drug candidates, mainly attributed to their innate pharmacological properties and favourable safety profiles.^15^ In this vast natural repository, 2,4‐ditert‐butylphenol (2,4‐DTBP), a lipophilic phytocompound,^16^, ^17^, ^18^, ^19^ stands out with its plethora of biological activities that span from antioxidant,^20^, ^21^ anti‐inflammatory^22^ and antimicrobial^23^, ^24^, ^25^, ^26^, ^27^ to notable anti‐cancer properties.^28^ However, a distinct knowledge gap persists in understanding how 2,4‐DTBP modulates the activities of Bcl‐2 and Survivin, especially in the context of CRC cell proliferation and apoptotic responses. In addressing this lacuna, the present study embarks on a groundbreaking journey, employing rigorous methodologies to elucidate the molecular interplay between 2,4‐DTBP and these pivotal antiapoptotic proteins in CRC. With a focus on uncharted territories, our research promises not only to contribute novel insights into CRC therapeutics but also to redefine the potential of naturally derived compounds in oncological applications.

## 2 MATERIALS AND METHODOLOGY

### 2.1 Molecular docking

In order to delve into the binding capabilities of 2,4‐DTBP with target proteins Survivin (PDB ID: 1F3H) and Bcl‐2 (PDB ID: 6GL8), we utilized molecular docking methodologies. The computational process was carried out using Autodock version 4.2.1, a widely accepted software known for its precision and robustness in predicting ligand‐protein interactions.^29^ To ensuring the reliability and reproducibility of the results, three independent docking runs were performed. Each run generated a total of 50 solution poses, operating within a population size of 500. The key parameters set for the docking experiments were 2,500,000 evaluations and a maximum of 27,000 generations, ensuring thorough sampling of the conformational space. The other software settings were maintained at their default values to maintain the standard protocol. Subsequent to the docking procedure, root mean square deviation (RMSD) clustering was employed to interpret and group the obtained docking poses. The poses were re‐clustered employing three distinct clustering tolerances, viz., 0.5 Å, 1 Å and 2 Å. This strategy aids in pinpointing the most favourable ligand pose cluster, gauged by the lowest energy score and maximum population.

The binding pocket for Survivin was ascertained from prior literature.^30^ Key residues involved in ligand binding include LEU‐96, PHE‐93, VAL‐89, LEU‐98, PHE‐101, LEU‐104, LYS‐15, PHE‐86, LEU‐102, LEU‐6, TYR‐10, LEU‐14, PHE‐13, ARG‐18, LEU‐87 and ILE‐74. Accordingly, a grid box encapsulating this binding pocket was defined with centre coordinates *X* = 31.4487, *Y* = 2.1169, *Z* = 16.214 and spanning dimensions of *X*:30, *Y*:25, *Z*:24. Conversely, the binding cavity for Bcl‐2 was derived from the structural database (https://www.rcsb.org/structure/6GL8).^31^ The residues forming this cavity were delineated based on a 3 Å radius encompassing the co‐crystallized ligand. The resultant grid box for Bcl‐2 docking was positioned at *X* = 14.09, *Y* = 15.47, *Z* = 15.48, with dimensions set at *X*:3.53, *Y*:0.58, *Z*:12.41. This rigorous and comprehensive methodology was essential for revealing the intricate details of how 2,4‐DTBP interacts with both Survivin and Bcl‐2, shedding light on its potential therapeutic implications.

### 2.2 Molecular dynamics simulations

Molecular dynamics (MD) simulations are carried out for the complexes between Bcl‐2 and Survivin with 2,4‐DTBP using Desmond 2020.1 from Schrödinger, LLC. The simulation set‐up utilized the reliable OPLS‐2005 force field, renowned for its accuracy in modelling biological systems.^31^, ^32^ An explicit solvent model was employed, and TIP3P water molecules filled a salvation box of dimensions 10 Å × 10 Å × 10 Å.^33^ The physiological ionic environment was mimicked using a 0.15 M NaCl solution, with Na+ ions neutralizing any net charge. To ensure system stability and optimal conformational sampling, an NVT ensemble equilibrated the protein–ligand complexes for 10 ns. This was followed by a 12‐ns NPT ensemble equilibration and minimization. The simulations incorporated the Nose‐Hoover chain coupling scheme for temperature regulation^34^ and utilized the Martyna–Tuckerman–Klein chain coupling approach for pressure control.^35^ Key parameters like the electrostatic interactions were computed using the particle mesh Ewald method.^36^ For accuracy in capturing dynamic interactions, bonded forces were calculated at every 2‐fs time step by the RESPA integrator. The overall simulation was conducted for a substantial duration of 100 ns, and essential parameters such as RMSD, Rg, RMSF, H‐bonds, salt bridges and SASA were tracked to monitor system stability and interactions.

### 2.3 Cell lines and reagents

Since HCT116 cells have been used in a range of biological investigations, including colorectal cancer proliferation, therapeutic research and drug screening, we used this colorectal cancer cell line for our research along with a normal human fibroblast cell line, GM00637. We got the colorectal cancer cell line (human; HCT116) from NCCS, India, and normal human fibroblast cell line (GM00637) from Jadavpur University, West Bengal, India. Normal and cancer cells were cultured in DMEM (HiMedia) with FBS (10%) (HiMedia) and 2% antibiotic (Penstrep; HiMedia) in a CO_2_ incubator (Esco Scientific; at 37°C in a 5% CO_2_ environment). SRL Pvt. Ltd., India supplied the crystal violet. The drugs 2,4‐DTBP (Sigma‐Aldrich; Product No.: 137731) and 5‐fluorouracil (5‐FU) (Sigma‐Aldrich; Product No.: F6627) were used in this study.

### 2.4 MTT assay

The proliferation of HCT116 and GM00637 cells after treatment with 2,4‐DTBP was determined by MTT assay, as we described earlier.^37^ Cells were treated with or without 2,4‐DTBP and 5‐FU and incubated for 24 h. A 0.5 mg/mL concentration of MTT solution (100 μL/well) was added following incubation. At 570 nm, the absorbance was recorded using a plate reader (Thermo Scientific, USA).

### 2.5 Clonogenic assay

A total of 3 × 10^3^ HCT116 cells in each well of a six‐well plate were seeded, and the clonogenicity assay was performed according to the standard protocol.^38^ 2,4‐DTBP‐treated and vehicle‐controlled colonies were stained for 30 min with 0.5% crystal violet (SRL Pvt. Ltd., India) and washed with double distilled water. Images of each well were taken, and ImageJ software was used to count the colonies.

### 2.6 Cell migration assay

A uniform monolayer of HCT116 cells in 35 mm culture plates was scratched with a 10 μL microtip, treated with/without the IC_50_ concentration of 2,4‐DTBP and kept in the incubator for 24 h. The bright field images (0 and 24 h) of the same treated and vehicle‐controlled scratched area were taken by an inverted microscope (ZEISS Axio).^39^

### 2.7 AO/PI and DAPI fluorescent staining assay

The AO/EB (AO:EB = 1:1) and DAPI fluorescent staining procedures were performed to investigate the 2,4‐DTBP‐induced chromatin condensation in HCT116 colorectal cancer cells according to the standard protocols.^40^, ^41^

### 2.8 DNA fragmentation assay

The nuclear DNA damage caused by 2,4‐DTBP was studied using a DNA fragmentation assay.^42^ DNA isolated from the vehicle‐controlled and 2,4‐DTBP‐treated HCT116 cells was electrophoresed on a 1.5% agarose gel to separate the bands. The ChemiDoc™ MP imaging system (Bio‐Rad, USA) was used to capture images of the gel.

### 2.9 Scanning electron microscopic (SEM) study

HCT116 cells cultured on glass coverslips were treated with or without the IC_50_ dose of 2,4‐DTBP for 3 h. Following treatment, the cells were washed in PBS and fixed at room temperature for 30 min in 2.5% glutaraldehyde (HiMedia; diluted in PBS). Postfixation, the cells were rinsed three times for 10 min with PBS. For lipid fixing, 1% osmium tetroxide (OSO_4_) was applied to the fixed cells for 1 h.^43^ After washing, graded ethanol was used to dry the cells (20%, 30%, 40%, 50%, 60%, 70%, 80% and 90%, once for each and twice in 100% for 10 min each). After being dehydrated with graded alcohol, the cells were then placed in tetramethylsilane [Si(CH_3_)_4_] overnight to complete the drying process. The next day, the samples were prepared for imaging by SEM (Carl ZEISS Sigma 300 VP).

### 2.10 Annexin V/PI flow cytometric assay

Treated (IC_50_ concentration of 2,4‐DTBP) and untreated (vehicle‐controlled) HCT116 cells were fixed at room temperature for 10–15 min with formaldehyde (4%). After fixation, the cells were kept on ice for 5–10 min. The cells were incubated for 10 min in a solution containing 5 mg/mL BSA in PBS after they had been washed and resuspended in the same solution. The exposed phosphatidylserine residues of cells were tracked using FITC‐Annexin V staining using the protocol outlined in the ‘Apoptosis kit’ (Molecular Probes). Flow cytometry was used to figure out how many cells were positive for Annexin V and PI alone or together. For dot blot analysis, Cell Quest Pro software (BD BioSciences, USA) was used.^44^

### 2.11 Cell‐cycle assay

HCT116 cells (50 × 10^3^ cells/35 mm culture plate) were seeded, and the following day, cells were treated with or without the IC_50_ concentration of 2,4‐DTBP for 24 h, trypsinized, and washed three times with cold PBS. After discarding the PBS, 70% ethanol was added drop by drop with moderate vortexing of the tube containing the cell palate to fix the cells and stored overnight at 4°C. The alcohol was discarded from each vial after centrifugation (3000 rpm) for 15 min at 4°C. Following a PBS wash, cells were stained for 30 min in the dark with a PI/RNAase solution. FACS Celesta (Becton‐Dickinson, Franklin Lakes, NJ) was used to detect cell cycle distribution, and FCS Express software was used to evaluate the resulting data. The intensity of the labelled cells' fluorescence is proportional to the amount of DNA they have.^45^

### 2.12 Western blot analysis

The western blot study was performed according to the standard protocol.^45^ SDS‐PAGE separated equal amounts of protein from 2,4‐DTBP‐treated and vehicle‐controlled HCT116 cells, which were transferred to a nitrocellulose membrane, and subsequently processed to detect the target protein. Primary antibodies against Bcl‐2 (CST 15071; Cell Signaling Technology, USA), Survivin (CST 2808; Cell Signaling Technology, USA), Caspase‐3 (BB‐AB0243; Bio Bharati Life Science Pvt. Ltd., India) and α‐tubulin (CST 2144; Cell Signaling Technology, USA; used as a loading control) were used to incubate the membranes. A secondary antibody coupled with horseradish peroxidase (Abcam, USA) was used to incubate the blots for 2 h at room temperature. The bands representing the individual proteins were seen using a ChemiDoc XRS System.

### 2.13 Cellular bioenergetics study

After counting the HCT116 cells with a TC‐10 cell counter (Bio‐Rad, Hercules, CA, USA), 1 × 10^4^ cells were seeded in each well and incubated for 24 h in a CO_2_ incubator. Following attachment, the cells were treated with/without the IC_50_ concentration of 2,4‐DTBP for 3 h. The same treatment was repeated three times. The bioenergetics study was performed by an Agilent Seahorse extracellular flux analyzer (XFe24; Seahorse Bioscience, Billerica, MA, USA) according to the standard protocol.^44^, ^46^

### 2.14 ProTox study

The OSIRIS Property Explorer was used to analyse pharmaceutically essential aspects of a natural chemical (2,4‐DTBP) swiftly; it predicts all‐natural compounds' Absorption, Distribution, Metabolism and Elimination (ADME) properties (https://www.cheminfo.org/Chemistry/Cheminformatics/Property_explorer/index.html). ProTox‐II (https://tox‐new.charite.de/protox_II/), a free in silico toxicity prediction web server, projected 2,4‐DTBP's toxicity profile. The PubChem Name of the drug was entered into the ProTox‐II graphical user interface for toxicity computation using machine learning algorithms‐based models that comprised acute (oral) and organ (liver) toxicities, toxicological endpoints, pathways and targets. The webserver's prediction types, training/test set compounds, cross‐validation, descriptors, and techniques were used without modification.^47^, ^48^

### 2.15 Statistical analysis

Graphs were made using GraphPad Prism‐8.2.1 (GraphPad Prism, RRID: SCR_002798) as well as Excel software (Microsoft Excel‐2019). Experiments were conducted three times, and the results of these experiments were reported as the mean ± SD of three data sets. A two‐tailed paired *t*‐test was performed when the number of groups was two to calculate the *p*‐value using the SPSS (IBM SPSS Statistics 25) software, which reflects the significance of differences between distinct sets of experimental data. *p* < 0.05, *p* < 0.01 and *p* < 0.001 are denoted by *, ** and ***, respectively.

## 3 RESULTS AND ANALYSIS

### 3.1 Molecular Docking

**FIGURE 1:**
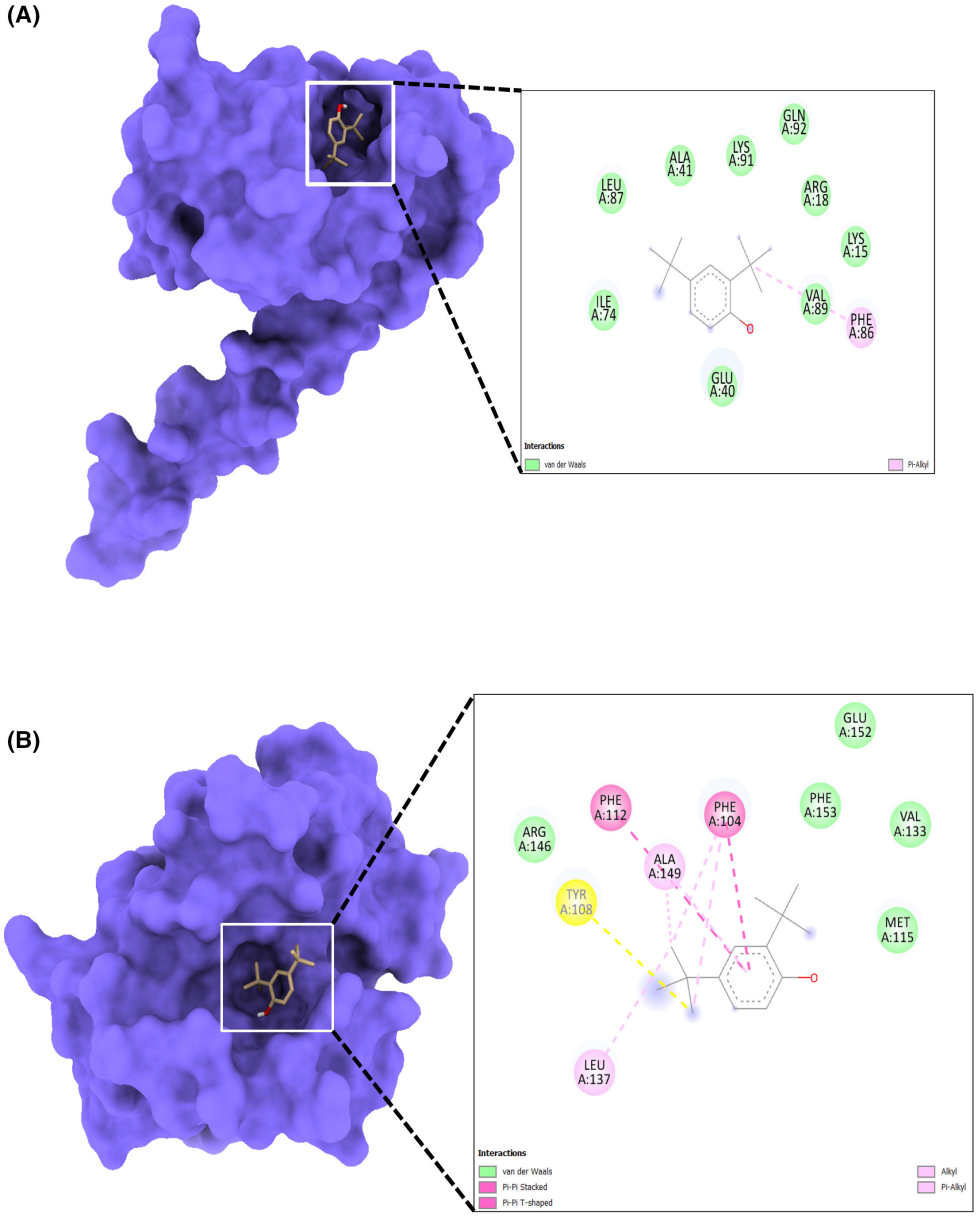
Molecular docking study: Surface view of the best pose of (A) Bcl‐2 + 2,4‐Di‐tert‐butylphenol and (B) Survivin+2,4‐Di‐tert‐butylphenol complexes displaying the surface view on the left panel and 2D interaction profile of the ligand with binding cavity residues.

In molecular docking analysis, the X‐ray crystal structures of proteins Bcl‐2 and Survivin have interacted with 2,4‐DTBP. During docking analysis, the highest population of ligand 2,4‐DTBP poses with Bcl‐2 belongs to the 0.25 Å RMSD cluster having the lowest binding energy Δ*G* = −9.8 kcal/mol has been considered for further analysis. Out of 50, 31 generated ligand pose populations fall within this cluster, ranging from 9.1 to 9.8 kcal/mol binding energies. The complex with the lowest binding energy exhibited pi–pi stacking with Phe104 and Phe112 residues of the binding cavity of Bcl‐2, while Ala149 was involved in pi–alkyl interaction with the ligand (Figure 1A). Met115, Val133, Arg146, Glu152 and Phe153 residues are involved in van der Waal's interaction (Figure 1A). Survivin interaction with 2,4‐DTBP exhibited the lowest binding energy Δ*G* = −5.6 kcal/mol and formed a pi–alkyl interaction with Phe86 residue (Figure 1B). This complex is further analysed for molecular dynamics simulation studies in order to analyse the complex's stability. The negative score of docking output signifies the free energy of binding (Δ*G*). The more negative Δ*G* indicates more efficient binding of the ligand with the respective proteins.

### 3.2 Molecular dynamics simulation

**FIGURE 2:**
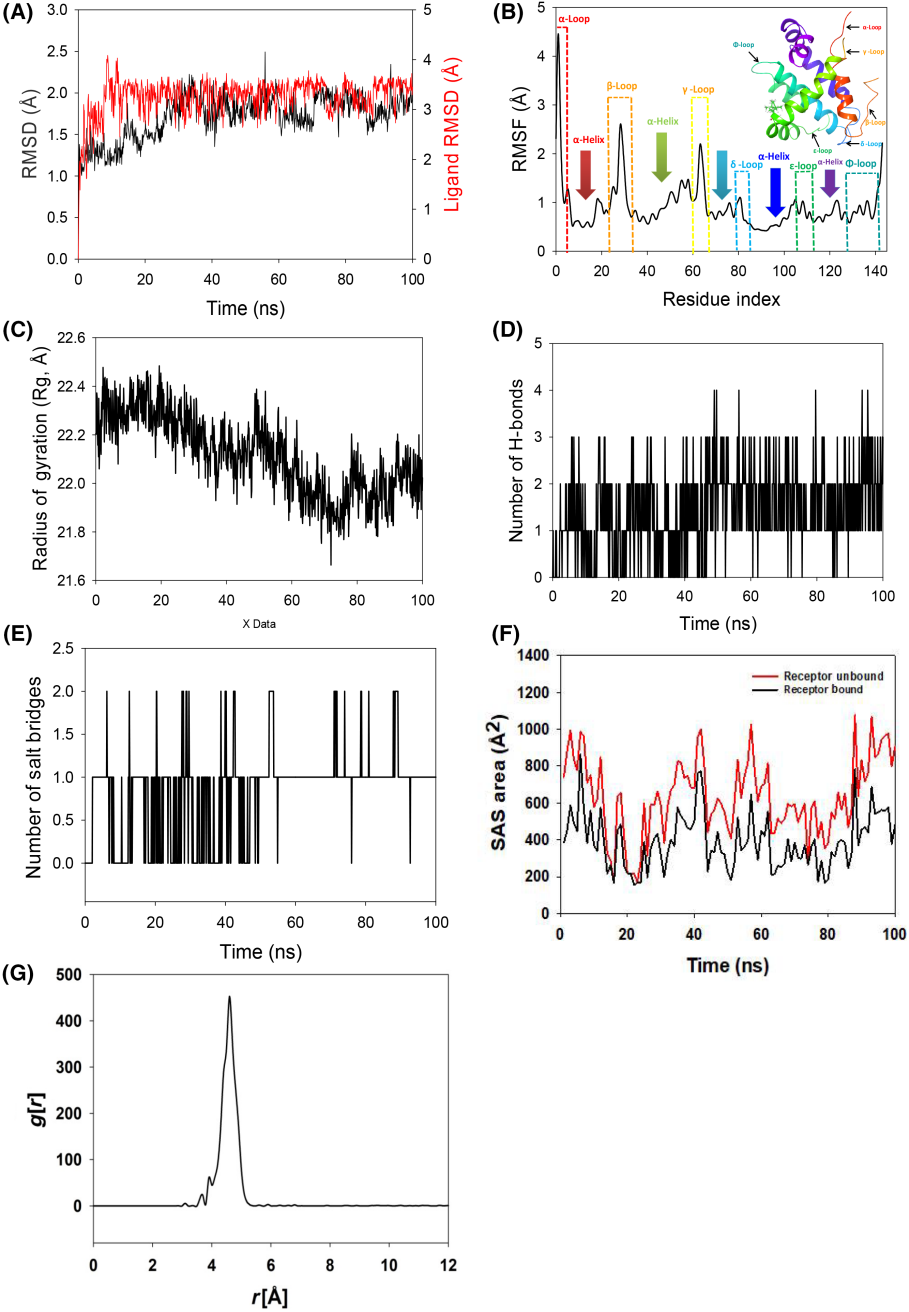
Molecular dynamics simulation analysis of 100 ns trajectories and radial distribution function of Bcl‐2 bound with 2,4‐DTBP complex: (A) Cα‐backbone of Bcl‐2 + 2,4‐DTBP‐ligand, (B) RMSF of Cα‐backbone of Bcl‐2 bound with 2,4‐DTBP‐ligand, (C) Radius of gyration (Rg) of Cα‐backbone of Bcl‐2 bound with 2,4‐DTBP (D) Formation of hydrogen bonds in Bcl‐2 bound with 2,4‐DTBP complex, (E) Numbers of salt bridge formation between and 2,4‐DTBP, (F) Solvent accessible surface area of Bcl‐2 bound with 2,4‐DTBP complex and (G) Radial distribution function (RDF) indicating the distance *g*[*r*] of 2,4‐DTBP ‐ligand from binding cavity residues of Bcl‐2.

To assess the convergence and stability of Bcl‐2 + 2,4‐DTBP and Survivin+2,4‐DTBP, molecular dynamics and simulation (MD) investigations were performed. Comparing the root mean square deviation (RMSD) values for simulations of 100 ns revealed stable conformation. The Cα‐backbone of Bcl‐2 that was bound to 2,4‐DTBP‐ligand displayed an RMSD of 1.9 Å (Figure 2A). While the ligand RMSD of 2,4‐DTBP‐ligand is depicted as 2.0 Å (Figure 2A). Stable RMSD plots during simulation indicate convergence and stability. So, it can be hypothesized that 2,4‐DTBP bound to Bcl‐2 is quite stable due to the ligand's high affinity. The plot for root mean square fluctuations (RMSF) indicates the residual fluctuations due to conformational variations into different secondary structures. Here, the RMSF plot displayed six distinct loops formed due to fluctuating residues, while high fluctuations were observed among 1–5, 23–35 and 60–67 residual positions (Figure 2B). The highest fluctuating peak comprised of 4.5 Å coined as α‐loop, β‐loop 3.2 Å and γ‐loop 3.1 Å, respectively (Figure 2B). Other three loops formed between 79–84 residues (δ‐loop), 106–112 residues (ε‐loop) and 134–143 residues (Φ‐loops) (Figure 2B). Rest small spikes conformed into α‐helices. Therefore, the protein Bcl‐2 has significant flexibility to conform to specific secondary structures in order to accommodate the ligand. The radius of gyration (Rg) quantifies the protein's compactness. Bcl‐2 Cα‐backbone bound to 2,4‐DTBP‐ligand demonstrated a decrease in radius of gyration (Rg) from 22.3 to 22.01 Å (Figure 2C). A significantly decreasing gyration (Rg) indicates that the ligand‐bound protein is in a highly compact orientation. The high number of hydrogen bonds between the protein and ligand is indicative of strong engagement and high complex stability. During the 100 ns simulation, the number of hydrogen bonds between Bcl‐2 and 2,4‐DTBP was significant (Figure 2D). Bcl‐2 and 2,4‐DTBP‐ligand exhibit an average number of consistent hydrogen bonds (average three numbers) (Figure 2D). Salt bridges are generated between nearby oppositely charged residues and serve a crucial role in protein stability.^49^ The average number of salt bridges formed between Bcl‐2 and 2,4‐DTBP‐ligand in this study (Figure 2E). Similar patterns were observed in both ligand‐bound and unbound states of solvent accessible surface area (SASA) after Rg analysis. Figure 2F clearly shows that in the unbound state of the 2,4‐DTBP‐ligand to the receptor, the protein Bcl‐2 displayed a large solvent accessible surface area (Figure 2F, red). The SASA value decreased when attached to the 2,4‐DTBP‐ligand compared to the unbound condition (Figure 2F, black). Overall, the Rg study shows that when ligands bind to proteins, they become more compact and less flexible. The interaction between the ligand and active site amino acid residues centre of mass is analysed by studying the radial density function (RDF). RDF finds the probability of distances between two particles from their centres. In this study, the distance of the ligand 2,4‐DTBP from the key binding residues of Bcl‐2 was detected after a pro‐simulation study, as shown in Figure 2G. The significance *g*[*r*] values indicated in the plot signify good interaction of the 2,4‐DTBP‐ligand with the residues, where the highest probability of finding the ligand near the residues was measured to be 4.5 Å (Figure 2G).

**FIGURE 3:**
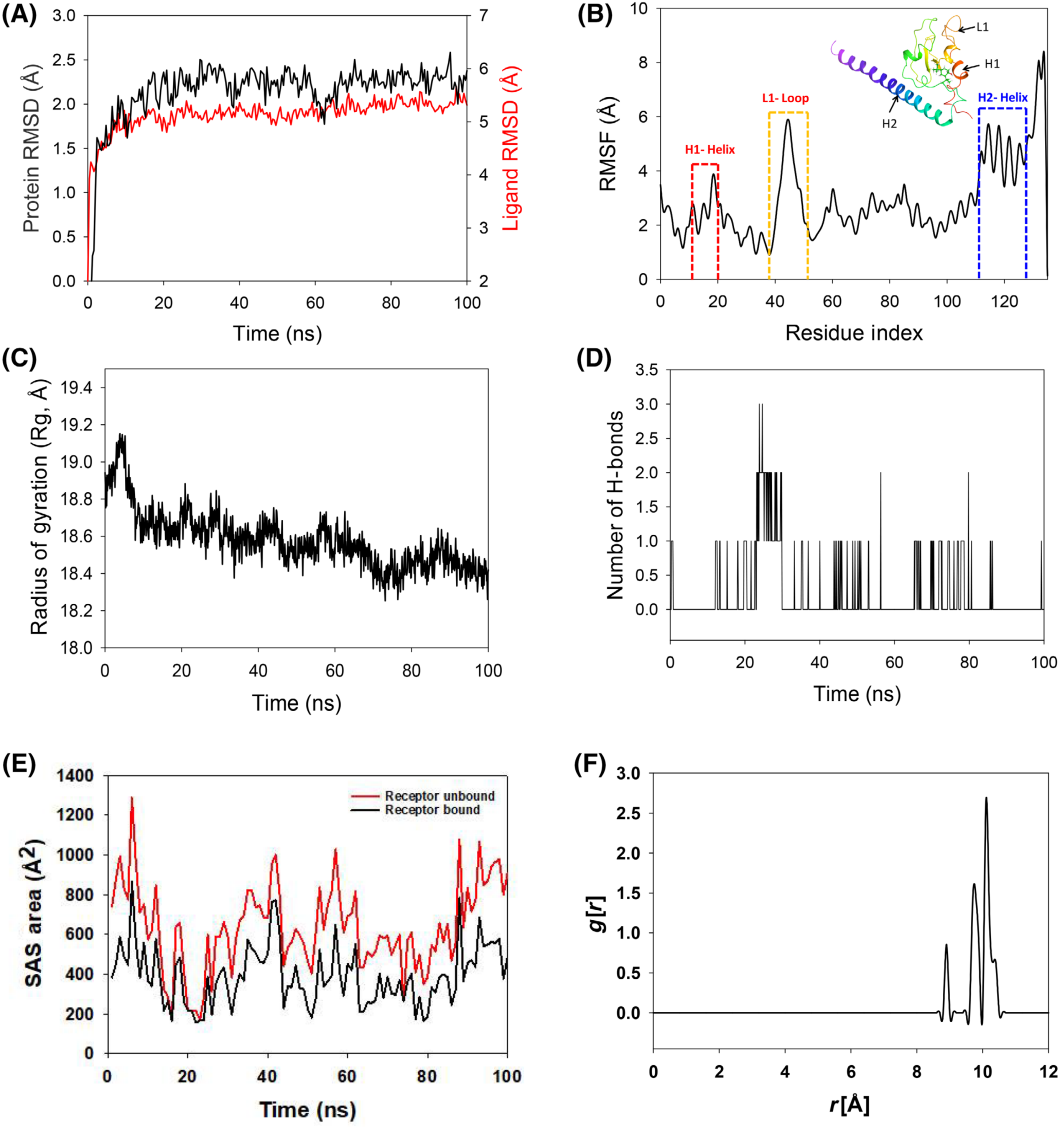
Molecular dynamics simulation analysis of 100 ns trajectories and radial distribution function of Survivin bound with 2,4‐DTBP complex: (A) Cα‐backbone of Survivin+2,4‐DTBP‐ligand, (B) RMSF of Cα‐backbone of Survivin bound with 2,4‐DTBP‐ligand, (C) radius of gyration (Rg) of Cα‐backbone of Survivin bound with 2,4‐DTBP, (D) formation of hydrogen bonds in Survivin bound with 2,4‐DTBP complex, (E) solvent accessible surface area of Survivin bound with 2,4‐DTBP complex and (F) radial distribution function (RDF) indicating the distance *g*[*r*] of 2,4‐DTBP‐ligand from binding cavity residues of Survivin.

The RMSD of Cα‐backbone of Survivin bound to 2,4‐DTBP‐ligand exhibited a deviation of 2.1 Å (Figure 3A). The ligand RMSD of 2,4‐DTBP‐ligand is depicted as 5.2 Å (Figure 3A). Stable RMSD plots during simulation indicate convergence and stability. Therefore, it can be hypothesized that 2,4‐DTBP bound to Survivin is quite stable due to the ligand's high affinity. The plot for root mean square fluctuations (RMSF) indicates the residual fluctuations due to conformational variations into different secondary structures. Here, the RMSF plot displayed 1 distinct loop and 2 helices formed due to fluctuating residues, while high fluctuations were observed among the 15–20 (H1‐helix), 38–56 (L1‐loop) and 115–125 (H2‐helix) residual positions (Figure 3B). The highest fluctuating peak comprised 5.8 Å for L1 loop, 3.8 Å for H1 helix and 6 Å for H2 helix, respectively (Figure 3B). Rest small spikes conformed into α‐helices. Therefore, the protein Survivin has significant flexibility to conform to specific secondary structures in order to accommodate the ligand. The radius of gyration (Rg) quantifies the protein's compactness. Survivin Cα‐backbone bound to 2,4‐DTBP‐ligand demonstrated a decrease in radius of gyration (Rg) from 18.85 to 18.4 Å (Figure 3C). Significantly decreasing gyration (Rg) indicates that the ligand‐bound protein is in a highly compact orientation. The high number of hydrogen bonds between the protein and ligand is indicative of strong engagement and high complex stability. During the 100 ns simulation, the number of hydrogen bonds between Survivin and 2,4‐DTBP was significant (Figure 3D). Survivin and 2,4‐DTBP‐ligand exhibit an average number of consistent hydrogen bonds (Average 1 numbers) (Figure 3D). Similar patterns were observed in both ligand‐bound and unbound states of solvent accessible surface area (SASA) after Rg analysis. Figure 3E clearly shows that in the unbound state of 2,4‐DTBP‐ligand to the receptor, the protein Survivin displayed a large solvent accessible surface area (Figure 3E, red). The SASA value decreased when attached to the 2,4‐DTBP‐ligand compared to the unbound condition (Figure 3E, black). Overall, the Rg study shows that when ligands bind to proteins, they become more compact and less flexible.

Usually, water bridges play a significant role in the presence of water as a solvent to hold the ligand in proper proximity to the binding residues of proteins, as confirmed by the RDF study. In the 2,4‐DTBP–Survivin interaction, the ligand distance from the key residues of Survivin exhibited much lesser (Figure 3F) compared to Bcl‐2. The lesser distance indicates less interaction, which might be due to the absence of salt bridges between Survivin and 2,4‐DTBP.

### 3.3 Molecular mechanics generalized born surface area (MM‐GBSA) calculations

Using the MD simulation trajectory, the binding free energy and other contributing energy in the form of MM‐GBSA are determined for the Bcl‐2 + 2,4‐DTBP and Survivin+2,4‐DTBP complexes. The results (Table 1) suggested that the maximum contribution to ΔGbind in the stability of the simulated complexes was due to ΔGbindCoulomb, ΔGbindvdW, ΔGbindHbond and ΔGbindLipo, while ΔGbindCovalent and ΔGbindSolvGB contributed to the instability of the corresponding complexes. Bcl‐2 + 2,4‐DTBP complex has comparatively higher binding free energies dGbind = −54.85 ± 6.79 kcal/mol higher than Survivin+2,4‐DTBP complex dGbind = −32.36 ± 1.29 kcal/mol (Table 1). These results demonstrated that Bcl‐2 + 2,4‐DTBP and Survivin+2,4‐DTBP have the potential to form stable protein‐ligand complexes with a high affinity for binding to the protein, efficacy in binding to the target protein and the ability to efficiently attach to the target protein.

**TABLE 1:** Binding free energy components for the Bcl‐2 + 2,4‐DTBP and Survivin+2,4‐DTBP complex calculated by MM‐GBSA.

Energies (kcal/mol)	Bcl‐2 + 2,4‐DTBP	Survivin+2,4‐DTBP
ΔGbind	−54.85 ± 6.79	−32.36 ± 1.29
ΔGbindLipo	−16.18 ± 1.04	−12.02 ± 2.94
ΔGbindvdW	−46.19 ± 2.18	−26.91 ± 2.28
ΔGbindCoulomb	−25.27 ± 6.20	−15.71 ± 2.10
ΔGbindHbond	−1.93 ± 0.34	−1.53 ± 2.34
ΔGbindSolvGB	32.34 ± 3.34	41.56 ± 1.14
ΔGbindCovalent	5.83 ± 4.51	7.31 ± 2.21

### 3.4 Time series analysis of Bcl‐2+2,4‐DTBP and Survivin+2,4‐DTBP complex

**FIGURE 4:**
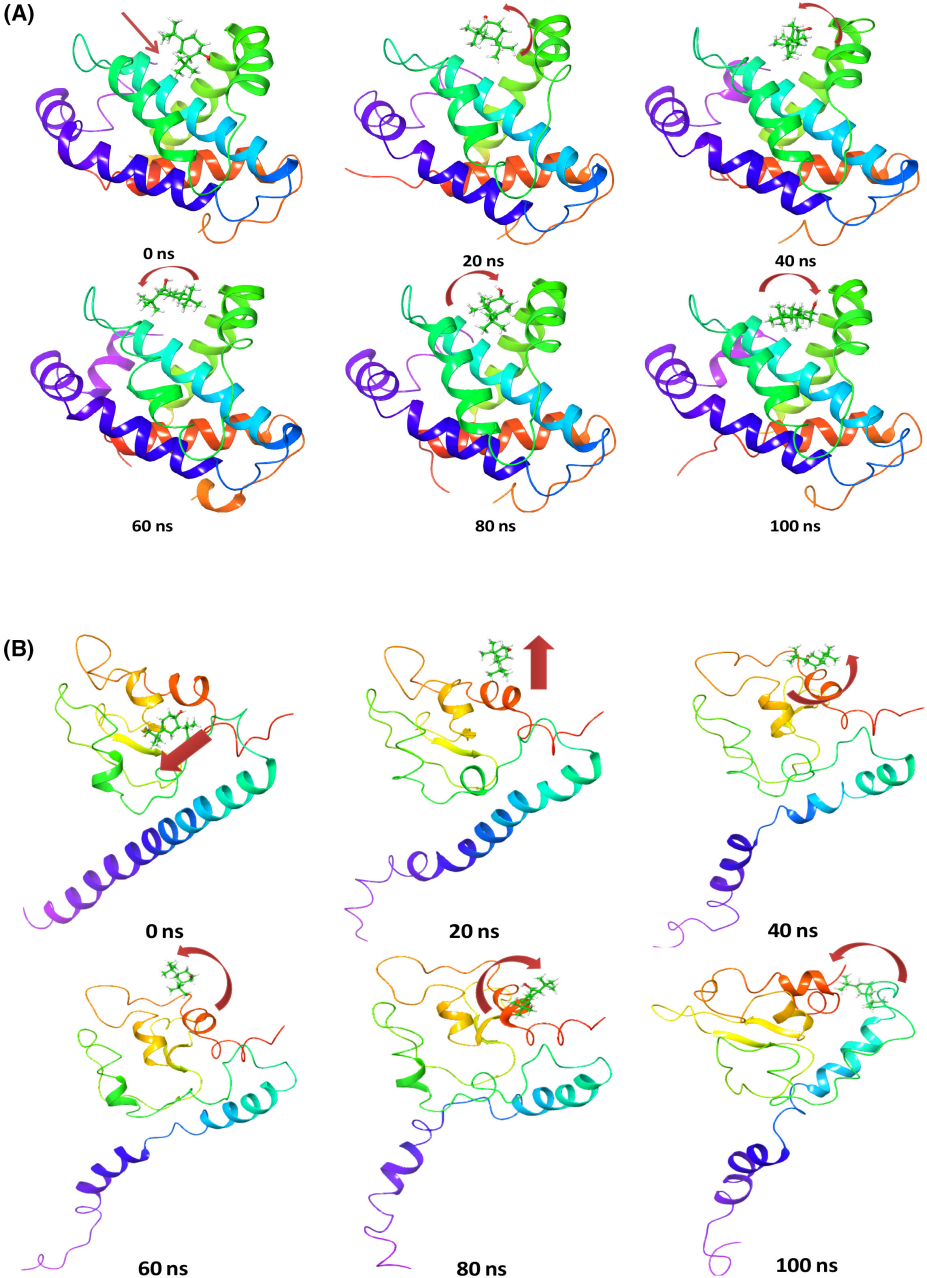
Time series analysis of Bcl‐2 + 2,4‐DTBP and Survivin+2,4‐DTBP complex: Time series analysis of the MD trajectory in order to understand the behaviour of the (A) Bcl‐2 + 2,4‐DTBP complex and (B) Survivin+2,4‐DTBP complex.

Time series analysis of MD simulation frames of Bcl‐2 + 2,4‐DTBP from the beginning of simulation (0 ns), 20, 40, 60, 80 and 100 ns was recorded and represented in Figure 4. At the beginning of the simulation, the ligand 2,4‐DTBP exhibited a linear arrangement (Figure 4A, arrow), while at 20 ns, 2,4‐DTBP moved and bent a little from its initial pose. These angular movements were observed through the simulation to orient the ligand 2,4‐DTBP for better accommodation within the binding pocket (Figure 4A). Time series analysis of MD simulation frames of Survivin+2,4‐DTBP from the beginning of simulation (0 ns), 20, 40, 60, 80 and 100 ns was recorded and represented in Figure 4B. At the beginning of simulation, the ligand 2,4‐DTBP exhibited a linear tilted arrangement (Figure 4B, arrow), while at 20 ns 2,4‐DTBP moved outward linearly from its initial pose. At 40–100 ns, the pose of the ligand is arranged to occupy different dimensions. These angular movements were observed through the simulation to orient the ligand 2,4‐DTBP for better accommodation within the binding pocket (Figure 4B).

### 3.5 2,4‐DTBP‐induced cytotoxicity

**FIGURE 5:**
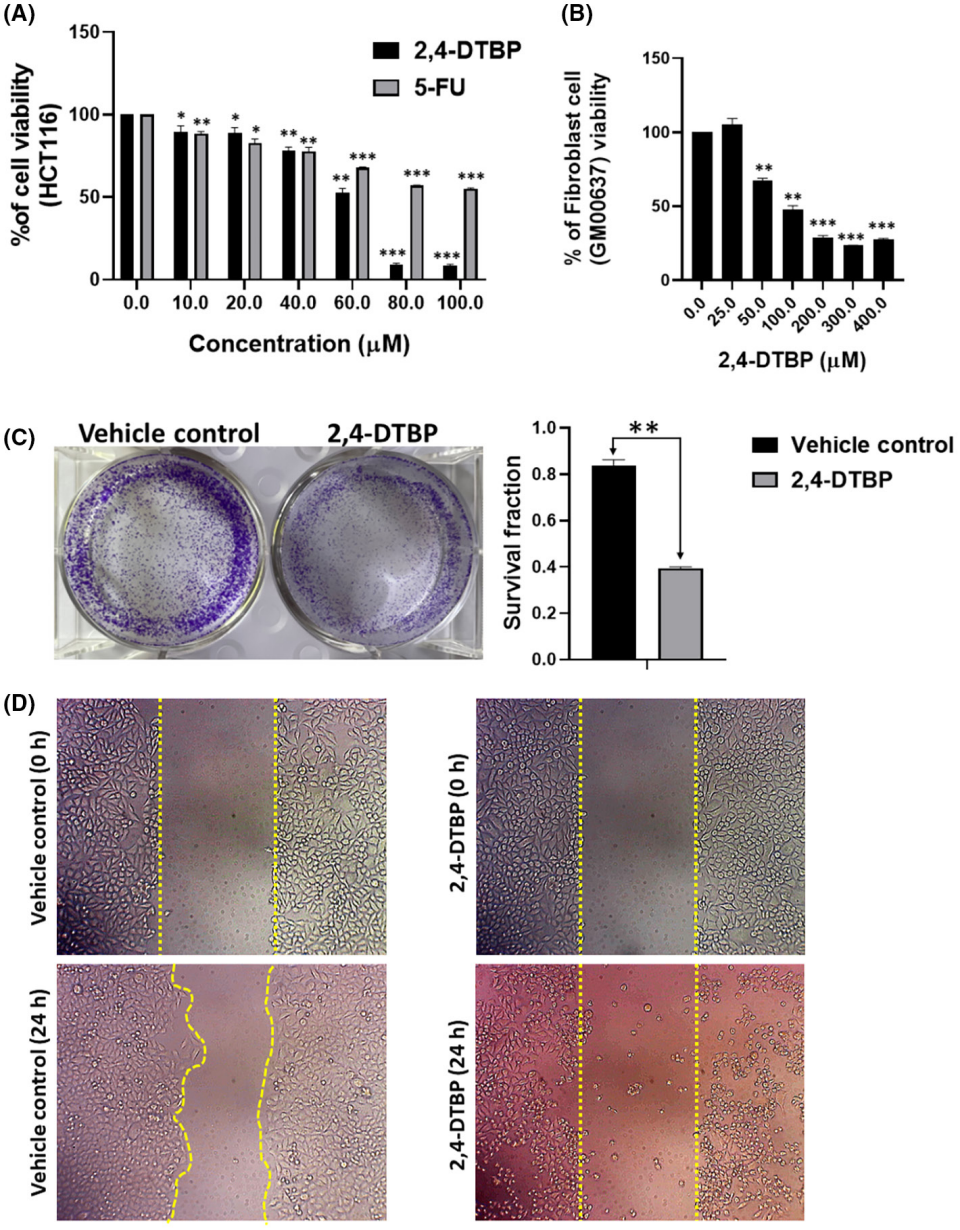
Cytotoxic, anti‐clonogenic and anti‐migrating potential of 2,4‐DTBP. (A, B) Cell viability assay (MTT assay). (A) The bar graph demonstrates the percentage of viability of HCT116 colon cancer cells at different concentrations of 2,4‐DTBP and the positive control, 5‐FU. (B) The bar graph demonstrates the percentage of viability of human fibroblast cells (GM00637) at different concentrations of 2,4‐DTBP. (C) HCT116 cells treated with (IC_50_) or without (vehicle control) 2,4‐DTBP. The treated group of cells shows significant anti‐clonogenic potential compared to the vehicle‐controlled group of cells. (D) Scratched areas of untreated (vehicle‐controlled) and 2,4‐DTBP‐treated HCT116 colon cancer cells at 0 and 24 h, indicating inhibition of migration of HCT116 cells upon treatment. Data are presented as mean ± SD (A, B and C graphs). *p < 0.05, **p < 0.01 and ***p < 0.001 vs. vehicle control, which was determined by a two‐tailed paired *t*‐test.

The antiproliferative effect of 2,4‐DTBP in HCT116 (human colorectal cancer) cells and GM00637 (human normal fibroblast) cells was determined by exposing both cell lines to a series of 2,4‐DTBP concentrations for 24 h (Figure 5A,B). HCT116 cells were also treated with the conventional anti‐cancer drug 5‐FU (used as a positive control). An in vitro cytotoxicity assay (MTT) was used for 24 h to measure the percentage of cell viability with or without (vehicle control) the drugs. The estimated IC_50_ value of 2,4‐DTBP in HCT116 cells was 57.044 ± 0.32 μM. However, it showed lower toxicity on the GM00637 cells (90.84 μM ± 4.28 μM) compared to the colorectal cancer cells. 5‐FU exhibited a higher IC_50_ (>100 μM) than 2,4‐DTBP in colorectal cancer cells (Figure 5A).

### 3.6 2,4‐DTBP inhibited the clonogenic potential

Using a clonogenic test, we validated the impact of 2,4‐DTBP on the ability of each HCT116 cell to form colonies. When 2,4‐DTBP was used at the IC_50_ concentration for 24 h, the number of colonies was significantly reduced in the experimental group compared to the untreated vehicle control group (Figure 5C).

### 3.7 2,4‐DTBP inhibited the cell migration

The impact of 2,4‐DTBP on cell migration was evaluated by a migration assay. Once a scratch was made using a 10 μL microtip into a monolayer of HCT116 cells, the cells were incubated with the IC_50_ concentration of 2,4‐DTBP for 24 h, and images of the scratched site were captured between 0 and 24 h. Comparing the gap size of treated and untreated (vehicle‐controlled) wounds (0 and 24 h), it was revealed that the gap size of the untreated scratch reduced after 24 h, whereas, in case of treated scratch, 2,4‐DTBP prevented the cell migration after 24 h of treatment (Figure 5D). So, the results suggest that 2,4‐DTBP is a potential anti‐migrating agent.

### 3.8 2,4‐DTBP‐induced morphological changes indicating apoptosis

**FIGURE 6:**
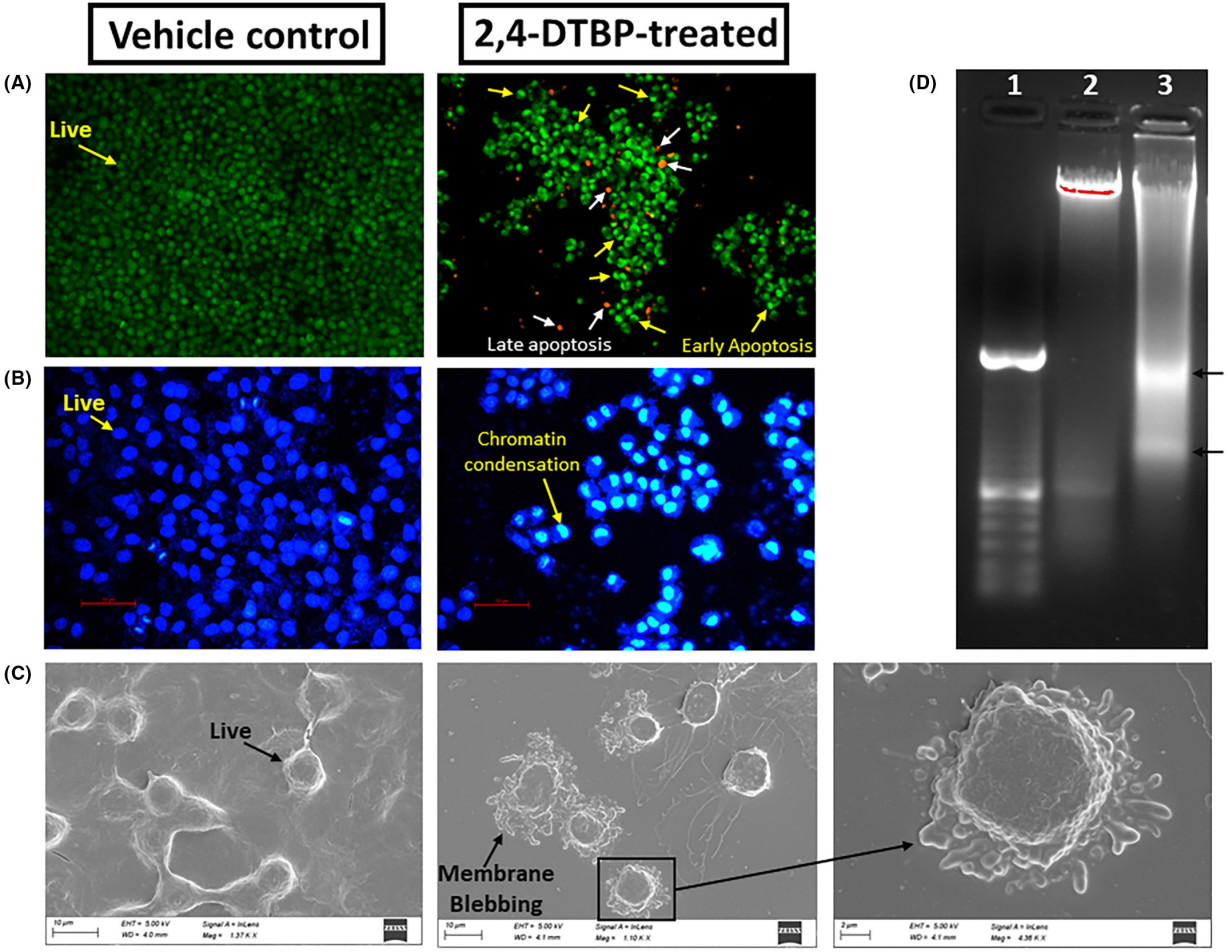
Apoptotic morphological changes induced by 2,4‐DTBP in HCT116 cells. (A) Acridine orange/Ethidium bromide (AO/EB) fluorescent staining shows a group of untreated (vehicle‐controlled), uniformly green‐stained live HCT116 colon cancer cells and a group of 2,4‐DTBP‐treated HCT116 cells with yellow‐green (early apoptosis) or orange (late apoptosis) coloured condensed chromatin. (B) DAPI fluorescent staining represents an untreated (vehicle‐controlled) uniformly blue‐stained live cell population and a group of 2,4‐DTBP‐treated HCT116 cells with a condensed nucleus, indicating apoptosis. (C) Scanning electron microscopic images show normal morphology of the vehicle‐controlled (untreated) HCT116 cells, whereas, 2,4‐DTBP‐treated HCT116 cells show prominent membrane blebbing, indicating apoptosis. (D) Represents the agarose gel image of a DNA fragmentation assay. Lane: 1 represents the DNA ladder (100 bp), Lane: 2 represents the isolated DNA of untreated HCT116 cells and Lane: 3 represents the isolated DNA of HCT116 cells treated with 2,4‐DTBP.

The acridine orange/ethidium bromide (AO/EB) (AO:EB = 1:1) fluorescent staining procedure was used to find out if alterations caused by apoptosis took place in the nucleus of HCT116 colorectal cancer cells. Results were seen after 24 h of incubation. After 24 h, the majority of the HCT116 cells in the vehicle‐controlled group fluoresce a light‐green colour due to AO nuclear staining (Figure 6A), but cells treated with the IC_50_ concentration of 2,4‐DTBP for 24 h fluoresce a yellow‐green or orange‐coloured condensed chromatin, which represents early and late apoptosis, respectively (Figure 6A).^40^ DAPI (4′,6‐diamidino‐2‐phenylindole) may also be used to detect apoptosis by observing nuclear alterations as it binds specifically and firmly to the minor groove of the adenine‐thymine sections of DNA. The distinct nuclear morphology of apoptotic cells, such as chromosome condensation and disintegration, aids in the detection of DAPI‐stained apoptotic cells.^41^ In response to 2,4‐DTBP (treated with the IC_50_ concentration for 24 h) treatment, chromatin condensation in HCT116 cells was observed using DAPI staining, which is a hallmark of apoptosis, whereas vehicle‐controlled cells had a uniformly blue‐stained nucleus (Figure 6B). Plasma membrane blebbing is also a morphological sign of cells going through late‐stage apoptosis. A bleb is an unusual bulge in a cell's plasma membrane brought on by localized cytoskeleton‐to‐plasma membrane dissociation. An apoptotic cell's plasma membrane is severely degraded and lacks the integrity needed to maintain crucial transmembrane gradients.^50^, ^51^ Under a scanning electron microscope, treated HCT116 colorectal cancer cells (with the IC_50_ concentration of 2,4‐DTBP) showed prominent membrane blebbing that indicates late apoptosis, whereas the vehicle‐controlled cells did not show any such feature (Figure 6C). Fragmentation of DNA is another characteristic of late‐stage apoptotic cells.^52^, ^53^ To see whether 2,4‐DTBP may cause DNA fragmentation and consequently apoptosis, HCT116 cells exposed to 2,4‐DTBP (IC_50_ concentration for 24 h), tested for DNA laddering and observed using agarose gel electrophoresis, which exhibited a DNA fragmentation profile (Figure 6D) characteristic of apoptosis. However, untreated (vehicle‐controlled) cells showed no signs of nucleic acid fragmentation.

### 3.9 2,4‐DTBP‐induced apoptosis detected by annexin V/PI double staining

**FIGURE 7:**
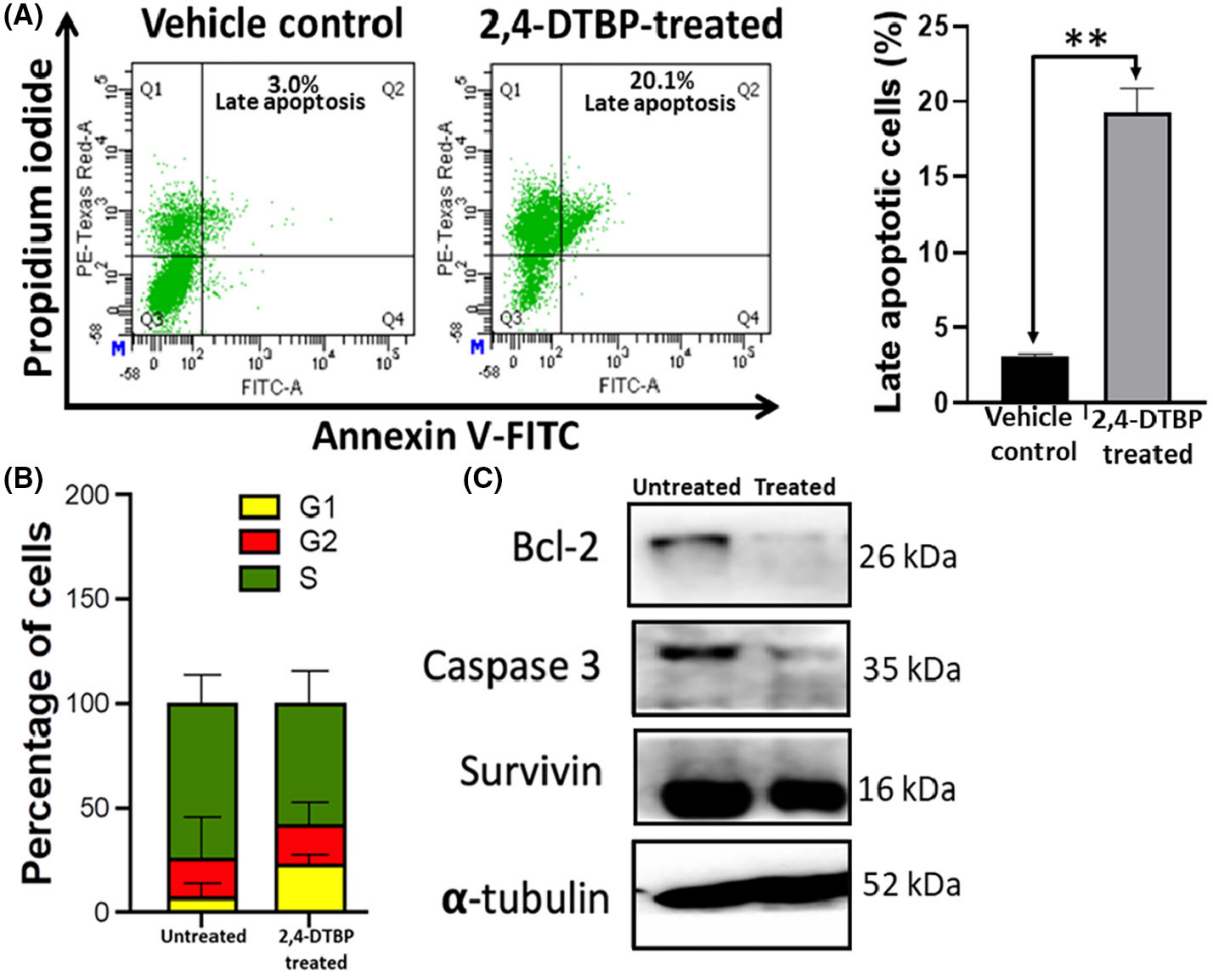
Detection of apoptosis by annexin V‐FITC/PI flow cytometric assay. (A) The effect of 2,4‐DTBP on apoptosis induction was determined by Annexin V‐FITC/PI flow cytometric assay. The bar graph represents the % of apoptotic cells in the experimental groups compared to vehicle‐controlled groups (an average of three sets). In this representative experimental group, 20.1% of treated cells showed late apoptosis (Annexin V+, PI+), compared to the vehicle‐controlled (untreated) cells (3.0% of cells showed late apoptosis). Cells were classified as healthy cells (Annexin V−, PI−), early apoptotic cells (Annexin V+, PI−), late apoptotic cells (Annexin V+, PI+) and damaged cells (Annexin V−, PI+). The error bar demonstrates the mean ± SD of three independent experiments; **p < 0.01 vs. vehicle control. (B) Induction of cell cycle arrest in HCT116 cells by 2,4‐DTBP. The bar graph represents the percentage of each phase of the cell cycle arrest in HCT116 cells, treated with or without 2,4‐DTBP. The percentage of each phase of the cell cycle was obtained using FCS Express software. (C) Western blot results show the expression of anti‐apoptotic Bcl‐2 and Survivin proteins along with Caspase‐3. α‐tubulin represents a loading control.

After observing the apoptotic morphology from the aforementioned assays, we further examined the potential of 2,4‐DTBP in inducing apoptosis in HCT116 colorectal cancer cells by using a flowcytometric assay with Annexin V and PI. An increase in the Annexin V and PI both positive cells from 3.1% (±0.17%) to 19.27% (±1.62%) was observed after 3 h of 2,4‐DTBP treatment with the IC_50_ concentration for 24 h (Figure 7A). So, ultimately, the flow cytometric assay results confirm the potential of 2,4‐DTBP to induce apoptosis in HCT116 cells.

### 3.10 2,4‐DTBP‐induced cell cycle arrest

Disruption of normal cell cycle progression is the most often recognized event in the emergence of cancer. Cell cycle arrest is a mechanism through which some phytochemicals have been shown to prevent the development of cancer cells.^54^ To better comprehend the mechanism of action of 2,4‐DTBP on HCT116 cells, the cell cycle distribution of HCT116 cells was assessed using flow cytometry. Comparing untreated (vehicle‐controlled) cells to those treated with the IC_50_ concentration for 24 h, we found that most of the treated cells underwent an arrest in the cell cycle's G1 phase compared to the untreated group. This data suggest that the reduction in cell proliferation and viability of 2,4‐DTBP‐treated HCT116 cells may have been caused by the triggering of cell cycle arrest at distinct stages of the cell cycle (Figure 7B).

### 3.11 2,4‐DTBP‐induced alteration of protein expression

Results of western blot showed that in treated (IC_50_ concentration for 24 h) HCT116 cells, anti‐apoptotic proteins Survivin and Bcl‐2 had lower levels of expression compared to vehicle control. 2,4‐DTBP also activated the pro‐Caspase‐3 into active Caspase‐3. Here, α‐tubulin is used as a loading control (Figure 7C).

### 3.12 2,4‐DTBP‐induced mitochondrial stress

**FIGURE 8:**
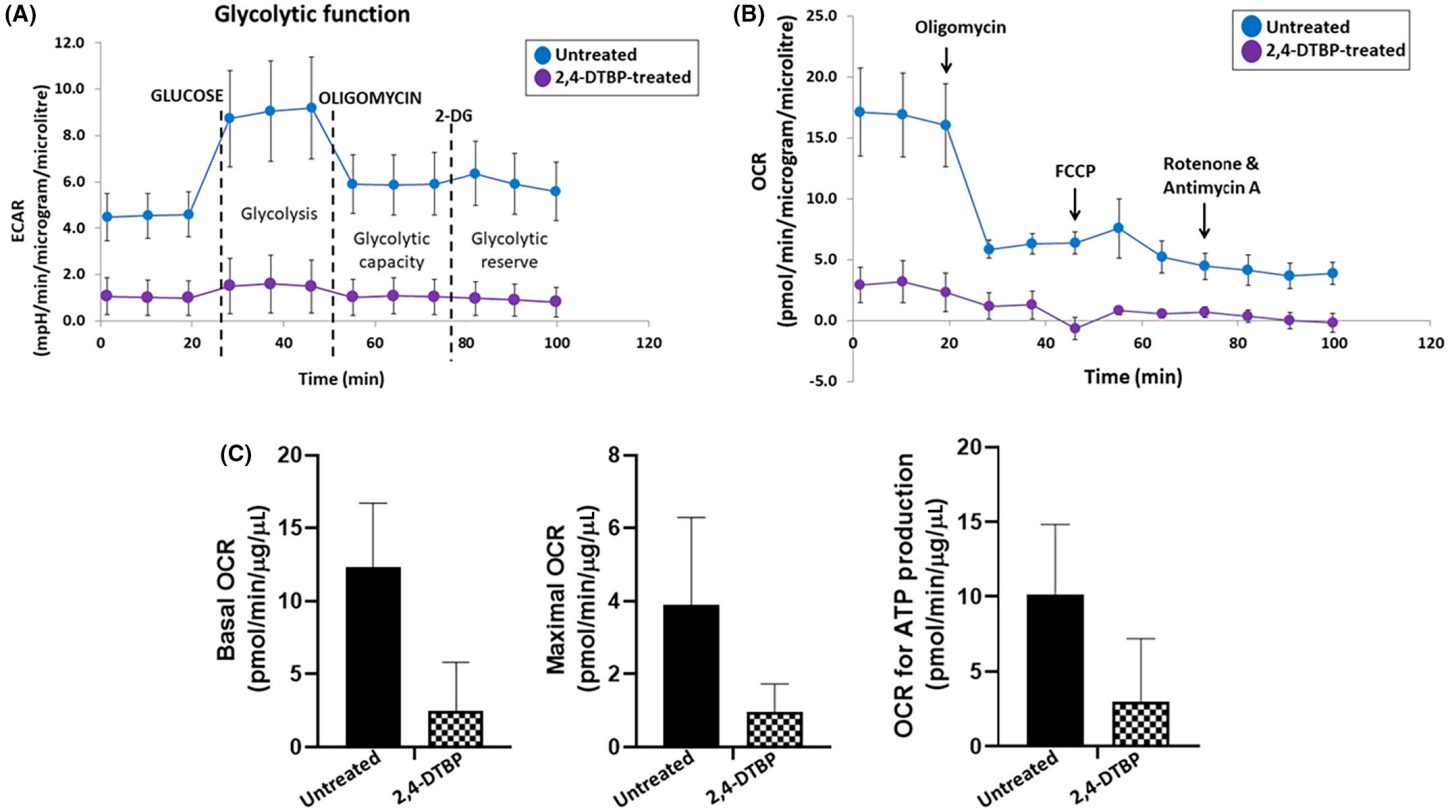
Cell Mitostress assay. (A) The extracellular acidification rate (ECAR) of untreated (vehicle‐controlled) and 2,4‐DTBP‐treated HCT116 cells, (B) mitochondrial oxygen consumption rate (OCR), (C) basal respiration, maximal respiration and ATP production.

Mitochondria play a key role in deciding whether a cell will continue to live or die (apoptosis).^55^ Therefore, we concentrated on cellular bioenergetics to comprehend the survival strategies of treated (IC_50_ concentration of 2,4‐DTBP for 3 h) and untreated HCT116 colorectal cancer cells. An investigation of metabolic activity was done by the Agilent Seahorse extracellular flux analyzer (XFe24). The Extracellular Flux Analyzer is capable of measuring both the oxygen consumption rate (OCR), an indicator of mitochondrial respiration and the extracellular acidification rate (ECAR), an indicator of net proton loss during glycolysis, in a living cell at the same time.^56^ Many bioenergetic characteristics can be determined by monitoring the ECAR and OCR in response to 2,4‐DTBP.^56^, ^57^ The ECAR and OCR readings in each well were normalized to the total protein concentration (BCA protein assay, Pierce). A percentage of basal OCR is used for the ATP synthesis of the cells.^58^ The cell mitostress assay showed reduced ECAR (Figure 8A), mitochondrial OCR (Figure 8B), basal mitochondrial respiration (Figure 8C), maximal mitochondrial respiration (Figure 8C) and mitochondrial ATP production (Figure 8C) in 2,4‐DTBP‐treated HCT116 cells than in untreated vehicle‐controlled cells.

### 3.13 In silico prediction of ADME and toxicity of 2,4‐DTBP

The ligand structure was retrieved from the PubChem database. OSIRIS Property Explorer assessed the recruited natural chemical based on its ADME properties and toxicity profile. The synthesized compound was run through a drug‐likeness filter. The filter's requirements were molecular weight in the range of 160–480, number of hydrogen bond donors in the field of 0–7 and obeying Lipinski's rule indicating drug‐like properties of 2,4‐DTBP (Table 2).

**TABLE 2:** Predicted toxicity risks and predicted properties from OSIRIS Property Explorer.

*Predicted toxicity risks*	
Mutagenic	No
Tumorigenic	No
Irritant	No
Reproductive effective	No
*Predicted ADME properties*	
cLogP	4.48
Solubility	−3.64
Molweight	206.33
TPSA	20.23
Drug‐likeness	−5.83
H bond acceptor	1
H bond donor	1
Nb stereocenters	0
Nb rotatable bonds	2
Drug‐Score	0.40

**FIGURE 9:**
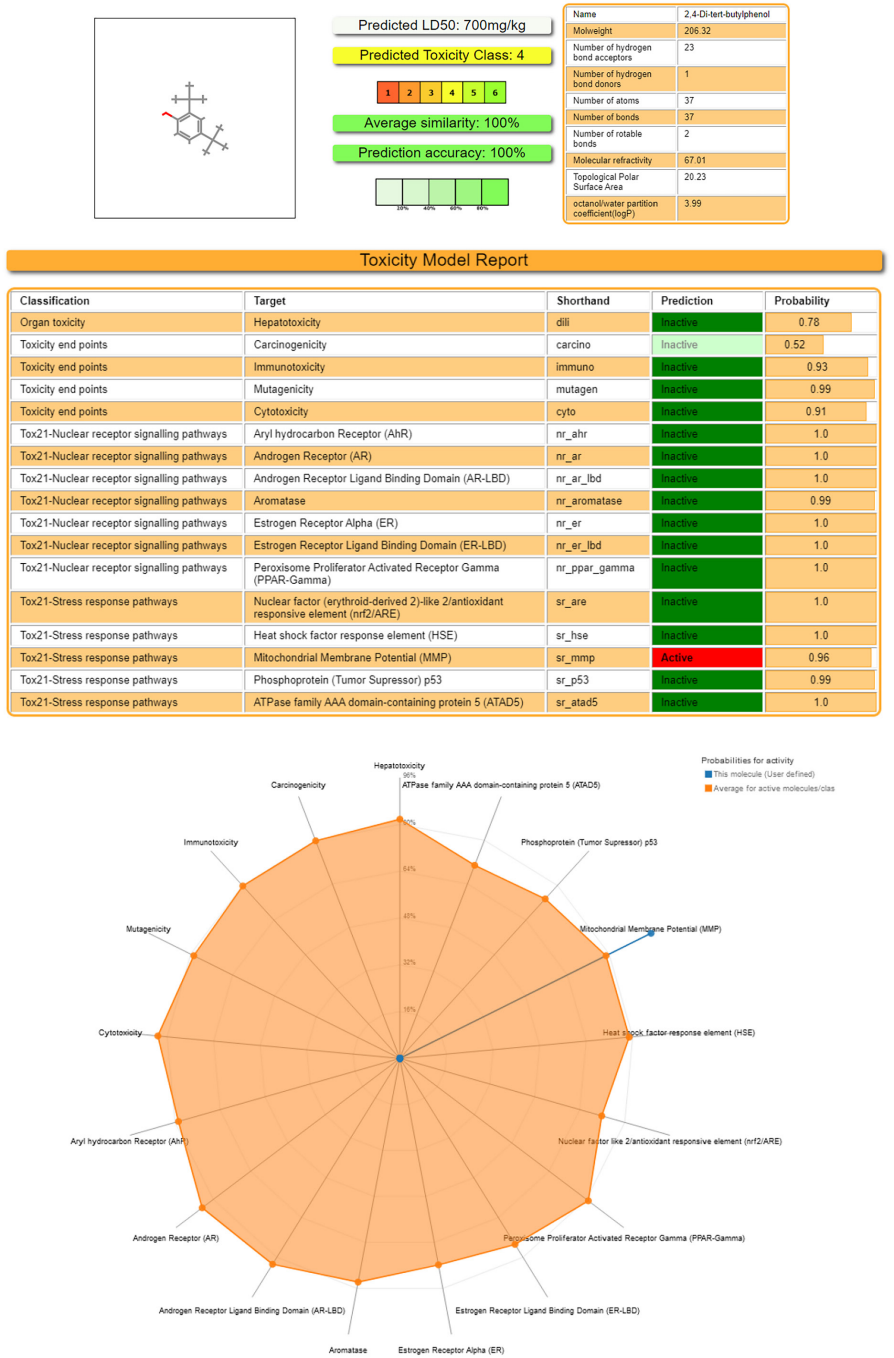
In silico evaluation of the toxicity of 2,4‐DTBP. (A) Oral toxicity prediction results; (B) Toxicity Model Report; (C) The toxicity radar chart. Evaluation processed by ProTox‐II server (https://tox‐new.charite.de/protox_II/).

Regarding acute oral toxicity, based on the ProTox results, Compound 2,4‐DTBP had an LD50 value of 700 mg/kg BW.^59^ Its predicted toxicity score is 4 (toxicity class: 4).^60^ So, it is much less toxic in nature. It shows activity on Tox21‐Stress response pathways, that is, on Mitochondrial Membrane Potential (MMP), which signifies induction of mitostress (Figure 9).

## 4 DISCUSSION

In maintaining the delicate equilibrium of cellular homeostasis, especially within the colorectal region, the balance between proliferation and apoptosis is pivotal.^61^ Disruption of this balance can be a driving force behind the evolution and progression of colorectal cancer (CRC).^61^ The presence of Survivin in CRC is strongly associated with the expression of Bcl‐2, though they exert different and non‐overlapping anti‐apoptosis mechanisms.^62^, ^63^ The membrane protein Bcl‐2, primarily located on the mitochondrial outer membrane, effectively prevents the release of cytochrome c, thus hindering apoptosis initiation.^64^ In contrast, Survivin blocks apoptosis by targeting the terminal effector caspase‐3.^65^ By inhibiting both Bcl‐2 and Survivin, there is an observed enhancement in radiosensitivity and chemoresistance in tumour cells, ultimately amplifying the therapeutic response against CRC.^62^ Given the reliance of CRC tumours on these anti‐apoptotic proteins throughout the disease's different stages, these proteins present themselves as potential therapeutic targets.^61^ As a result, a number of research groups have developed several inhibitors targeting Bcl‐2 and Survivin, however, compounds concurrently targeting both proteins remain scarce.^66^, ^67^, ^68^ Therefore, the present study primarily focused on examining the inhibitory capacities of 2,4‐DTBP against both Bcl‐2 and Survivin. Molecular docking and molecular dynamics simulations firmly established the significant binding potential of 2,4‐DTBP with survivin and Bcl‐2. To strengthen this molecular evidence, a series of in vitro assays were conducted.

Through cytotoxicity assays, it was ascertained that 2,4‐DTBP could limit HCT116 colorectal cancer cell proliferation in both time‐ and dose‐dependent fashions, demonstrating its heightened efficacy compared to 5‐FU, the conventional control. The capability of 2,4‐DTBP to impair the cell migration and colony‐forming ability of HCT116 cells was corroborated further by the cell migration assay and the clonogenic assay, respectively.

Morphological assessments of 2,4‐DTBP‐treated HCT116 cells showcased classical apoptotic features such as nuclear shrinkage, chromatin condensation and membrane blebbing. These observations were further fortified by DNA fragmentation patterns consistent with apoptosis.

The apoptosis‐inducing potential of 2,4‐DTBP in HCT116 cells was further confirmed by using Annexin V and propidium iodide (PI) staining (flowcytometric assay), which revealed a significant increase in apoptotic cells.

Furthermore, cell cycle analysis identified a predominant arrest at the G1 phase, pointing towards the influence of 2,4‐DTBP on CRC cell cycle dynamics. A comprehensive western blot analysis elucidated the decline in Bcl‐2 and Survivin levels post‐2,4‐DTBP treatment, alongside the activation of pro‐Caspase‐3, suggesting the involvement of mitochondrial pathway of apoptosis. This pathway's functionality is closely intertwined with mitochondrial ATP production and membrane stability. Assessments of mitochondrial bioenergetics in treated cells revealed decreased OCR and ECAR, indicative of attenuated mitochondrial respiration and net proton loss during glycolysis, respectively, potentially leading to mitochondrial stress. In light of its potent anti‐CRC activities, 2,4‐DTBP's toxicity profile was investigated in silico, revealing its non‐toxic nature across several crucial parameters, marking it as a promising candidate for future CRC therapeutic applications.

## 5 CONCLUSION AND FUTURE DIRECTIONS

The compound 2,4‐DTBP stands out in this research for its remarkable inhibitory potential against both Bcl‐2 and Survivin. Through rigorous molecular docking and in vitro assays, 2,4‐DTBP illustrated not just commendable binding efficacy but also an ability to impede CRC cell proliferation significantly. Its consequential impact on cell cycle dynamics, primarily inducing an arrest at the G1 phase, and its role in triggering apoptosis via the mitochondrial pathway, accentuate its therapeutic prospects. Given that Survivin and Bcl‐2 constitute two pivotal pro‐survival mechanisms fostering therapeutic resistance in cancer cells, our revelation that 2,4‐DTBP concurrently downregulates both proteins becomes particularly consequential. This discovery positions 2,4‐DTBP as a potential agent to enhance drug sensitivity in CRC cases that inherently resist or develop resistance to cancer therapeutics.

Furthermore, with the burgeoning interest in proteolysis‐targeting chimaeras (PROTACs) in the realm of precision therapeutics, the future might see an amalgamation of PROTAC principles with compounds like 2,4‐DTBP. By leveraging PROTACs' inherent capacity for selective protein degradation, we can potentially refine the specificity and potency of agents like 2,4‐DTBP, furthering their therapeutic response against CRC. In addition, chemical modification of the compound may generate derivatives with higher efficacy. Ultimately, this study uncovers the promising role of 2,4‐DTBP as a potent therapeutic contender for CRC management, alluding to a future where precision and efficacy in treatment paradigms for CRC are further enhanced.

## AUTHOR CONTRIBUTIONS

**Partha Saha:** Conceptualization (equal); formal analysis (equal); software (equal); writing – original draft (equal). **Mangala Hegde:** Data curation (equal); formal analysis (equal); methodology (equal); visualization (equal); writing – original draft (equal). **Kanak Chakraborty:** Data curation (supporting); formal analysis (supporting); methodology (supporting); validation (supporting); visualization (supporting); writing – original draft (supporting). **Achinta Singha:** Formal analysis (supporting); methodology (supporting); visualization (supporting); writing – review and editing (supporting). **Nobendu Mukerjee:** Data curation (equal); formal analysis (equal); methodology (equal); software (equal); validation (equal); visualization (equal); writing – original draft (equal). **Deepshikha Ghosh:** Formal analysis (equal); supervision (equal); validation (equal). **Ajaikumar B. Kunnumakkara:** Data curation (equal); validation (equal); writing – review and editing (equal). **Mohd Shahnawaz Khan:** Methodology (equal); software (equal); validation (equal); writing – original draft (equal). **Md Irshad Ahmad:** Formal analysis (equal); investigation (equal); visualization (equal). **Arabinda Ghosh:** Conceptualization (equal); formal analysis (equal); supervision (equal); writing – review and editing (equal). **Ajoy Kumer:** Formal analysis (equal); methodology (equal); writing – original draft (equal). **Samir Kumar Sil:** Conceptualization (equal); project administration (equal); software (equal); writing – review and editing (equal).

## CONFLICT OF INTEREST STATEMENT

The authors declare no conflict of interest.

## DATA AVAILABILITY STATEMENT

The data used to support the findings of this study are available from the corresponding author upon request.
